# The Invisible Disease: Making Sense of an Osteoporosis Diagnosis in Older Age

**DOI:** 10.1177/1049732311416825

**Published:** 2011-12

**Authors:** Joanne M. Weston, Emma V. Norris, Emma M. Clark

**Affiliations:** 1 Plymouth University, Plymouth, Devon, United Kingdom; 2 University of Bristol, Bristol, United Kingdom

**Keywords:** aging, health care screening, health care, primary, health care, users’ experiences, illness and disease, experiences, interpretative phenomenological analysis (IPA), medicine, older people, relationships, health care, women’s health

## Abstract

Osteoporosis (low bone density) is a potentially serious disease which mainly affects women older than 50 years. National screening programs for osteoporosis are being developed in the United Kingdom. It is important to assess the psychological experience of receiving a positive diagnosis from a population-based screening program so that psychological distress does not outweigh medical benefits. Little research has been conducted in this field. In our study, we explored the experience of being diagnosed with osteoporosis following screening. We interviewed 10 women aged 68 to 79 who were recruited from a population-based osteoporosis screening trial. Four themes emerged from our interpretative phenomenological analysis of the interviews: osteoporosis is a routine medical condition, lack of physical evidence creates doubt, the mediating role of medical care, and protecting the self from distress. Our findings emphasize the complexity attached to receiving a positive screening result. We suggest considerations for health care providers.

Osteoporosis (low bone density) is a major public health concern worldwide, affecting one in three women aged 50 or older (Kanis et al., 2004; Melton, Chrischilles, Cooper, Lane, & Riggs, 1992). It is serious in terms of its disabling nature, reduced quality of life, and fracture-related mortality rate (Lips et al., 1999; Nevitt et al., 1998; Poole & Compston, 2006; Roberto & Reynolds, 2001). Osteoporosis is referred to as “the silent disease,” because it is usually asymptomatic and therefore often not detected unless a woman has a fracture and it comes to the attention of physicians (National Institute for Clinical Excellence [NICE], 2008). As it worsens, women often suffer problems such as chronic pain, physical deformity because of curvature of the spine (kyphosis) following vertebral fractures, and emotional problems such as anxiety, depression, and low self-esteem (Gold, 1996, 2001; Lips, 2003; Silverman, 2005).

Despite these statistics, there is no national screening program for osteoporosis in the United Kingdom or in many other countries worldwide, and research suggests its detection rate is often low (Premaor et al., 2010). The available evidence indicates that early detection and treatment is important. For example, treating a woman with a vertebral fracture who is older than 65 can reduce her risk of having another fracture in the next 5 years from one in four to one in eight (Kaptoge et al., 2004). Effective treatment is available in the form of medication, calcium supplements, dietary changes, and weight-bearing exercise (Johnell & Hertzman, 2006). There are currently two United Kingdom clinical trials for osteoporosis screening: The Screening of Older Women for the Prevention of Fracture (SCOOP) trial (Medical Research Council, 2007) and the Cohort for Skeletal Health in Bristol and Avon (COSHIBA) study (UK Clinical Research Network, n.d.). These community-based trials aim to evaluate cost-effective methods of screening for fracture risk. Their main purpose is to measure the medical benefits of screening in terms of reducing future fractures, but it is also important to consider the psychological aspects of screening, because the advantages from screening should be greater than any psychological harm caused to participants (one of the screening program criteria set by the United Kingdom National Screening Council; Department of Health, 2000). However, little research has taken place into the psychological reaction to receiving a positive result from an osteoporosis screening program. Therefore, it is useful to draw on research for general health screening to gain a greater understanding of this area.

The psychological impact of health screening is greatly debated (Croyle, 1995). Adverse effects of receiving a positive result can include general anxiety, health anxiety, and depression (e.g., Croyle, Smith, Botkin, Baty, & Nash, 1997; Rimes & Salkovskis, 2002; Wiggins et al., 1992). Postscreening problems such as illness-related work absenteeism, psychosomatic symptoms, and negative intrusive thoughts have also been reported (Haynes, Sackett, Taylor, Gibson, & Johnson, 1978; Sachs, 1995; Shaw, Abrams, & Marteau, 1999; Stewart-Brown & Farmer, 1997).

The challenge for researchers in this field arises because not all health screening studies report negative psychological effects. The authors of two systematic reviews that explored the psychological impact of screening for a variety of health conditions reported that results were inconclusive, with some studies showing an emotional impact from screening—such as anxiety and depression—that was not reflected in other research (Heshka, Palleschi, Howley, Wilson, & Wells, 2008; Shaw et al., 1999). Type 2 diabetes screening research also provides mixed results. The authors of one review noted that there was no adverse psychological impact of receiving a positive screening result for this condition, although short-term anxiety was slightly raised in two studies (Adriaanse & Snoek, 2006). Despite these findings, other researchers raised concerns about participants’ frequent poor understanding of diabetes as a condition, their confusion over the meaning of elevated blood glucose levels (despite being given information about this), and their possible misinterpretation of its seriousness (Adriaanse, Snoek, Dekker, van der Ploeg, & Heine, 2002). These suggest that we need to consider the wider issues that might affect someone’s reaction to screening, because psychometric measures alone might not provide a complete picture.

Overall, there is currently no general consensus in the literature regarding the psychological effects of health screening, because it largely seems to be study- and condition-specific. Research into the psychological impact of receiving a positive result from osteoporosis screening has been largely neglected. Anxiety levels of participants were measured in one study (Rimes, Salkovskis, & Shipman, 1999). The researchers concluded that there was no increase in general or osteoporosis-related anxiety in either the high- or low-risk screening groups at 3 months postdiagnosis. Other researchers included the Stait Trait Anxiety Inventory (Spielberger, 1983) as one of their quality-of-life measures in research exploring the use of hormone replacement therapy (which is no longer used as a treatment for osteoporosis) in perimenopausal women (Torgerson, Thomas, Campbell, & Reid, 1997). They found no difference in anxiety levels between the screened high-risk group (*n* = 607) and the control group (*n* = 613) 2 years postrandomization. However, this questionnaire was administered only once, nearly 2 years postscreening; therefore, there was no baseline or intermediate measure with which to compare results.

Although the authors of these two studies (Rimes et al., 1999; Torgerson et al., 1997) have started to explore women’s psychological reactions to receiving a positive result from an osteoporosis screening program, the researchers have mainly focused on measuring levels of anxiety experienced by the participants. In addition, participants in the studies were, on average, younger than those targeted by population-based screening programs. A more recent study, carried out using qualitative methodology, could provide a greater understanding of the specific difficulties women might encounter when receiving and adapting to a positive result. Researchers explored the bone scanning experience of 16 healthy Danish women aged 61 to 63 (Reventlow, Hvas, & Malterud, 2006). The results showed that every woman experienced detrimental effects from seeing a visual image of her bone loss. Following diagnosis, the women reconstructed their views of their bodies, perceiving them as more fragile; were anxious about the consequences of osteoporosis; and placed unnecessarily self-limiting restraints on their lifestyle to reduce the perceived risk of fractures.

Even though the research by Reventlow et al. (2006) was a small-scale and country-specific study, these findings suggest that quantitative methods might not be sensitive enough to provide sufficient insight into the psychological processes that might follow a positive result. Given that anxiety levels fluctuate over time and are not found to be clinically significant in all participants, the research findings so far provide very little information about the factors that might influence individual reactions and adjustment. Theories tentatively suggested by osteoporosis researchers to date include the Cognitive Behavioral Model of Health Anxiety; optimistic biases and minimization in the form of minimizing the seriousness of a positive result (Rimes & Salkovskis, 2002; Rimes et al., 1999); the Health Belief Model (Wallace, Wright, Parsons, Wright, & Barlow, 2002); and illness-specific beliefs (Reventlow et al., 2006). These suggest that we potentially have relevant psychological theories and models that might help us to understand the adjustment process in more depth, and build our knowledge in this area.

As we have discussed, there are some gaps in the research literature. First, we know little about how people experience a positive result from a population-based screening program for osteoporosis. Second, our understanding of the factors that might contribute to either a positive or negative adjustment to diagnosis is limited. Thus, there is a pressing need for more research in this area, because it is vital that when policy makers and medical staff decide to increase the number of people undergoing osteoporosis screening each year, they consider the overall psychological impact and adjustment processes that follow a positive result (Peckham & Dezateux, 1998; Reventlow et al., 2006).

Qualitative methodology gives a direct voice to each participant rather than relying on group measures and implicit researcher assumptions about what the significant adjustment factors might be. Interpretative phenomenological analysis (IPA) provides an ideal methodology to examine personal experiences of osteoporosis screening in detail. With its idiographic and phenomenological underpinnings, it is used to explore, from an insider’s perspective, how each individual makes sense of his or her diagnosis and the meanings that he or she gives to it (Smith, Flowers, & Larkin, 2009). At the same time, the role of the researcher’s own beliefs, assumptions, and interpretations of the data are acknowledged, which creates a dynamic two-stage interpretative element to the process (Smith, 1996). In the current study, we aimed to explore women’s experiences of receiving and adjusting to a positive result from an osteoporosis screening. In this article we specifically focused on what a diagnosis of osteoporosis meant to individual women and how they made sense of it.

## Method

### Participants

We purposively selected participants from patients who were participating in a research trial for an osteoporosis screening procedure in the United Kingdom: the Cohort for Skeletal Health in Bristol and Avon (COSHIBA). Methodological details of COSHIBA have been published elsewhere (Clark, Gould, Morrison, Masud, & Tobias, in press) but, in brief, recruitment for this trial took place at 15 general practitioner (GP) surgeries within the Bristol area of southwest England. The COSHIBA researchers sent a letter to every woman aged 65 to 80 years, inviting them to be screened for osteoporosis. There were no exclusion criteria, and 3,200 women were recruited. The screening process consisted of an interview with a nurse, who recorded personal osteoporosis risk factors, height, and weight. These enabled risk scores to be calculated for each woman, and those who fell into the at-risk group had an X-ray of their back to look for evidence of a vertebral fracture. The fracture was used as a diagnostic marker to confirm the diagnosis of osteoporosis. Women visited their GP approximately 3 weeks later to receive their results, which were delivered verbally.

The current research was a substudy of the COSHIBA research trial. We selected participants from the group who had been diagnosed with osteoporosis and were taking a once-a-week dose of bisphosphonate medication and daily calcium tablets for osteoporosis, as prescribed by their GP, therefore forming a homogenous sample consistent with the philosophy of IPA (Smith et al., 2009). Studies in which an IPA approach is adopted focus on a rich, in-depth exploration of individual experiences, and detailed interpretations of those experiences therefore benefit from using small sample sizes (Smith et al.). Consistent with these principles, the final sample for our study consisted of 10 White British women aged 68 to 79. They had attended their initial screening appointment between 11 and 19 months prior to the substudy. We excluded participants with serious health conditions such as cancer. Many of the participants had minor, well-controlled health conditions such as hypertension and high cholesterol.

### Procedures

We obtained ethical approval from the Gloucestershire Research Ethics Committee, then wrote to 18 women, providing study details and issuing an invitation to participate in the study. Those who returned consent forms were invited to a tape-recorded interview in their GP surgery. Interview lengths ranged from 45 to 60 minutes. We used an interview guide to encourage a broad approach to discussing the experience of receiving a diagnosis of osteoporosis (Smith et al., 2009). The interview was designed to focus on open questions to deepen the interviewer’s understanding of women’s experiences and the meanings they attributed to them. We gave the women contact details for the United Kingdom National Osteoporosis Society at the end of the interview so they could speak to a specialist osteoporosis nurse if they needed help or advice. The interviews were transcribed by the first author, Joanne Weston, and all identifying information was changed or removed to ensure confidentiality.

### Analysis

Weston analyzed the interviews using IPA methodology as described by Smith et al. (2009). First, she read each transcript several times to become familiar with its content and made notes in the margin. These included initial thoughts, key phrases used by the women, specific use of language that captured the experience of having osteoporosis, observations, and possible interpretations. Emergent themes were developed from these descriptions and grouped into similar items. These were used to create superordinate themes that reflected the meaning and interpretation of the experience of being diagnosed with osteoporosis for each woman. As the analysis continued, we looked for similar themes in subsequent transcripts, but remained open to allowing new themes to emerge as interpretations facilitated other meanings to develop. When all of the transcripts had been analyzed, we examined similarities and differences between interviews. We identified patterns across transcripts, creating superordinate themes and subthemes which we felt reflected the shared experiences of the women as a group.

At all stages of the process, we cross-checked emerging ideas against the original transcripts to ensure that our interpretations were driven by and consistent with the women’s accounts, without allowing our own experiences and beliefs to dominate. The role of the researchers in the analysis was carefully considered because this is a key element of IPA (Smith et al., 2009). Keeping a reflective diary and having discussions with two research supervisors helped monitor this process. Initially, we carried an early expectation that women would be distressed by their diagnosis; this changed as the interviews progressed and the emerging data challenged this view.

## Results

The women described quickly reaching a position of acceptance, or degrees of acceptance, about their positive screening result. The four themes that emerged—osteoporosis is a routine medical condition, lack of physical evidence creates doubt, the mediating role of medical care, and protecting the self from distress—suggest that the women struggled to understand the meaning of their diagnosis and its implications for their (then) current and future health.

### Osteoporosis is a Routine Medical Condition

Women often made sense of their diagnosis by positioning it on their life trajectory. They seemed to interpret it as part of the natural aging process and used their basic knowledge of the disease to make sense of it as something that was not very serious.

#### Osteoporosis as natural aging

Seven women reached an understanding of their osteoporosis by relating it to their long lifespan to date. The women frequently expressed a sense of inevitability that the natural aging process would result in a deterioration of their health:

I imagine as you get older your bones aren’t as good as they were, because nothing is. Your muscles aren’t so strong, are they? In the same way, your veins and arteries don’t work so well, they get clogged up. So, I mean, it’s obvious your skeleton will also have a certain amount of damage. . . . The crumbly status of old age.

Osteoporosis was generally interpreted as being a common and inevitable medical condition for older people. As one woman described, “It’s just that with old age, your bones are getting brittle. I know all old people don’t get it, but the chances are you will.”

Women also seemed to make sense of osteoporosis by comparing it with other common age-related conditions such as high cholesterol and hypertension. There was a general belief that some ill health was normal in this age group, and that it was unrealistic to expect otherwise. Osteoporosis was often viewed as just another label to add to an ever-growing list. One woman spoke about her osteoporosis and other health problems, saying, “It all comes under one umbrella to me.” This did not mean that the women were not concerned about their health conditions; however, they accepted that they could not stop the aging process.

#### Osteoporosis is not very serious

Women tended to hold on to the idea that osteoporosis is a disease that is a relatively minor health concern. They often compared themselves to other people their age who had more life-threatening or life-limiting conditions such as cancer and dementia, seemingly using the comparison to gain a sense that osteoporosis was not very serious:

I’ve got friends who’ve got much worse things wrong with them. I mean, I could have lung cancer or dementia—those poor souls. Now that would be serious and something to worry about. But at least I can take these tablets and be all right. I think I’m lucky that I haven’t got anything much to worry about really.

The knowledge that women seemed to draw on to reach the conclusion that osteoporosis was not a serious disease was limited. Descriptions of how they understood osteoporosis as a disease were often brief. They were all aware that it meant that their bones were more fragile and that their fracture risk was increased; however, only a few women accurately described the potential progression of the disease in terms of its disabling nature, physical deformity, and chronic pain. Two of these women had first-hand experience of seeing a close relative with osteoporosis. Some women expressed confusion over whether the osteoporosis was only present in their spine or in other parts of their skeleton as well. As a result, the women’s understanding of osteoporosis often seemed based on inaccurate or incomplete information:

I think the crumbling starts, the deterioration of the bones, is most likely to be around the joints where they get wear and tear and that kind of thing. I think it’s to do with little crystals forming. . . . I presume osteoporosis is something like osteoarthritis.

### Lack of Physical Evidence Creates Doubt

Osteoporosis as a disease had little meaning for the women. They were often asymptomatic, and those who had some aches and pains saw this as synonymous with aging. There was a lot of not knowing—in terms of the accuracy, severity, and reality of the diagnosis. Women had to guess what the diagnosis meant, and their degree of bone loss. Others found it difficult to accept the accuracy of the diagnosis because they did not have any physical evidence to support it.

#### The invisible disease

An underlying theme that emerged for many women was the struggle to accept a diagnosis when they felt healthy and had no visible signs of disease. This meant they felt that they had to believe an abstract diagnosis, or they interpreted it as incorrect or insignificant. The absence of visual evidence created mixed reactions to the diagnosis among the women. Some women described this as a good thing; they did not want to be reminded that they had something wrong with them because this would make them worry more. Others said how difficult it was to know they had a disease that was invisible. One woman emphasized her struggle to understand her diagnosis, saying, “That’s the hard part because you can’t see anything. . . . I know I’ve got it, but I can’t see it.” For those women who gave very little meaning to the diagnosis, the lack of symptoms maintained their prediagnosis views of themselves as healthy people who had a strong skeleton:

I’ve fallen over so many times without breaking anything at all, ever, that I’ve always considered I’ve got very strong bones! [laughs] But I don’t like to say that to the doctor because they’d be disappointed, very disappointed, I think.

I’d be very surprised if it was anything to worry about. I feel reasonably supple and I can’t imagine being in that situation where it’s really bad. . . . So, I’m not sure there’s much wrong with me, really.

#### Lack of pain means it isn’t serious

A frequent shared experience among the women was the difficulty of understanding a diagnosis that had no pain attached to it. Many women believed that having no pain meant they did not need to worry much; they saw pain as a marker of the seriousness of a disease. One woman said, “I mean, serious illness come with a lot of pain, so that’s when you really need to worry, isn’t it?” At the time of the interviews, they were generally living their lives as they had prediagnosis, and were fairly pain-free, with the exception of the few women who had arthritis. The lack of pain seemed to reduce the level of concern that women attached to their diagnosis:

I thought it might be painful, but it isn’t really. So, there’s not much point worrying then, I don’t see. I mean, if you broke several bones and you kept on doing it, I can see the point of people saying, “Oh you’ve got something wrong, you must do something about it.” If they knew definitively that was going to happen then I could see the point, but I can’t see that anybody does.

I saw my mother crippled with terrible pain with her arthritis. Now that’s real suffering. This problem I can live with. I don’t have pain, it doesn’t affect me in any way, and all I have to do is take some pills. I don’t think there’s any reason to get myself worried about it really.

Overall, the majority of the women used their perceptions of their own bodies to gauge how serious the diagnosis might be, and how much anxiety they needed to attach to it. Lack of pain frequently influenced this, giving osteoporosis a fairly benign meaning:

If I was in a lot of pain then I’d know that I was in a really bad way, but I’m not, so osteoporosis can’t be that much of a serious problem. Otherwise it’d hurt, and I’d know beyond doubt that it was in my body, um, bones, and causing me lots of trouble.

### The Mediating Role of Medical Care

Every woman’s story included accounts about how she interpreted the role of medical care in relation to her osteoporosis. The women seemed to hold a degree of trust in their GP and had strong ideas about medication as being the solution for their bone loss.

#### Trust in the GP

Most of the women demonstrated significant trust in their GP, and this gave them reassurance. This trust seemed to be founded on their GP’s successful treatment of previous health problems. They often described the GP as an expert. One woman said, “I haven’t got a good enough brain to understand it . . . so I’ll leave that to people that know.” There was a sense that many women unquestioningly accepted their GP’s knowledge and ability to treat their osteoporosis:

I know the doctors can’t be wrong. I just trust my GP will give me medication to keep my bones strong. If the doctor’s giving it to me then it must be helpful. . . . Despite my initial shock, I just thought, “Obviously he can do something, so I don’t need to worry.”

This was strengthened by most women’s good relationships with their GP. They described their doctors as people who were skilled, reassuring, and caring. This trust seemed to provide women with a sense that osteoporosis did not have potentially serious consequences because their GP could understand and treat it. Even among the few women who had a poor relationship with their GP and distrusted or did not understand the accuracy of the diagnosis or the need for medication, medication compliance was reported as high:

If I’m given something, I do take it. I wouldn’t waste it, [but] if the National Health [Service] wants to save money then that’s where they could save some. I wouldn’t mind not having the medication. I don’t think it’s necessary. I think everything’s so overmedicated.

None of the women had sought additional details from their GP about osteoporosis or asked further questions about the meaning of the diagnosis for them personally, such as its severity or how it might affect them. Their comments carried an implicit assumption that the GP would tell them everything they should know. Many expressed surprise when asked if they had requested, or researched, more information about osteoporosis:

Should I have? I didn’t have a lot of information, I must admit. . . . I just accepted what she said. And, as I said, I have great faith in my doctor to tell me anything I need to know.

#### Medication as the solution

Taking tablets to manage or cure medical problems came across as being standard practice for the women. Medication was part of their daily routine, so taking a once-a-week tablet and daily calcium supplements was generally manageable. Although many of the women disliked the weekly tablet and experienced some side effects, such as indigestion, most of the women’s interviews evidenced a belief in the importance of medicine. Medication was reported as being the biggest mediating factor in managing women’s feelings about the diagnosis. Even those who said they were initially shocked reported that they felt better emotionally as soon as they started medication (which was usually the following day). Medication provided a feeling of safety and reassurance because it was interpreted as a solution for their bone loss, and many women believed that it would stop the disease progressing. Having medication to take seemed to allay any concerns about the possible future prognosis and consequences of having osteoporosis:

I’m on the medication so that will sort me out. I don’t need to worry, as long as I keep taking it. I’ve been away for the weekend and thought, “Oh God, I didn’t take that tablet,” so . . . I read the instructions and it said to catch up the next day. So, once I take this week’s, I’m all right, then I have to start again next Monday.

The women’s belief in medication as a solution was strong, even though many of them said they had not thought about how it might benefit them or asked their GP about how their medication would help with their osteoporosis:

I don’t know. Whether it’s preventing it getting worse quicker—which is what I hope it’s doing—stabilizing it, or slowing it down, or strengthening the rest of my bones, or something. . . . I just think, I’m taking the medication and hopefully that will keep it at bay.

The women’s interviews demonstrated an underlying belief that medication was the only thing that could definitely help their osteoporosis. There was a lack of awareness of the benefits of weight-bearing exercise and a calcium-rich diet on bone health. One woman said, “This is nothing I can do anything about, apart from medication.” Exceptions were found among the women with a family history of osteoporosis. One woman said, “I do find myself thinking about that a bit more now . . . I have milk with cereal and cups of tea. . . . I do some exercises.” However, most women relied solely on taking medication to help themselves. Some of the women described how they were probably already looking after their bones by keeping themselves healthy through eating a low-fat diet, not being overweight, and taking exercise such as swimming (a misconception).

### Protecting the Self From Distress

The women frequently emphasized how important it was for them not to dwell on their diagnosis or to worry about it. There was a sense that they needed to be seen as positive people who did not allow their thoughts to affect their mood, and who were able to keep life events in perspective.

#### The influence of the mind

The women clearly expressed an underlying belief that negative thinking or worry had the potential to affect both their physical and mental health. They asserted that if they spent too much time thinking about their osteoporosis then they could make it worse. They feared they would become too anxious about damaging themselves, and therefore become less active, or too depressed to motivate themselves to do the activities they enjoyed. This would then impact on their physical health:

I think the mind can be quite powerful and convincing about any situation, whether it’s right or not. If I got overparanoid, I’d be sitting around, whereas I’m busy. . . . It’s probably better for your bones if you keep moving, and it’s definitely better for your mind.

Women also seemed to interpret worrying thoughts as having the ability to create further health problems and possibly psychosomatic symptoms as well. One woman described this, saying, “You can exaggerate things, I think, very easily. You can give yourself pains by thinking about it. That’s really silly, isn’t it?” They often had strong views about the futility of ruminating on their health and creating unnecessary anxiety for themselves, so they often chose to prevent that line of thought:

I wouldn’t want to find myself worrying about it. I really wouldn’t want to. I wouldn’t want to feel it was having that effect on my life, so maybe, I suppose, that’s where the, “Don’t even go there,” you know?

#### Keeping things in perspective

For some women, worrying about osteoporosis in its current asymptomatic state seemed out of proportion when considered in relation to their history. Some women described having tough lives, such as major bereavements or an impoverished upbringing. There was also a sense that the women were able to continue to focus on their everyday lives as usual, and they often talked about how they were able to absorb themselves in normal activities, making sense of their diagnosis in relation to their wider world rather than giving it a significant position in their lives:

I just carry on as if I haven’t got it. I go out and go shopping. I go ballroom dancing once a week. I work around the house and garden, just ordinary life really, what I’ve always been used to.

One strategy the women described for keeping their diagnosis in perspective was living for the “here and now.” They seemed to give meaning to their diagnosis by concentrating on its current presentation, and not looking ahead into the future. Most of the women were able to accept their diagnosis but did not let it dominate their lives. Some of the women seemed to manage any anxiety about it by telling themselves that if their osteoporosis did get worse, then it would be in the distant future. For example, one woman said, “I’m 73 now, so I’ve got about another 10 years before I might get it really badly, that’s what I like to think. My sister was much older than me when she developed it.”

Although the women did not allow the diagnosis to intrude on their lives, they described themselves as being more sensible than they were previously. These minor adaptations allowed them to manage their increased fracture risk but still live as normal. They described taking extra precautions against falling, for example, when it was icy, and they asked for aids such as handrails:

I’m a little more careful in the garden, where I put my tools, where I put my weed bin so I don’t fall over it, things like that. We’ve got quite a large patio with quite a number of steps. I’ve had a handrail put there and I’m more careful coming down them, whereas I wasn’t before. . . . I’m just a little more alert to the dangers if you did fall.

## Discussion

In this study, we explored how women experienced and gave meaning to receiving a positive result for osteoporosis from a population-based screening program. Four themes captured similarities across the women’s accounts. These were: osteoporosis is a routine medical condition, lack of physical evidence creates doubt, the mediating role of medical care, and protecting the self from distress.

Researchers to date have mainly focused on anxiety levels of women following a positive result (Rimes et al., 1999; Torgerson et al., 1997). The findings from our study show that anxiety was either absent or short-lived. It seemed that anxiety levels were influenced by the core meanings that the women gave to the diagnosis, and how they made sense of it in relation to their current lives. This suggests that there are a complex set of beliefs which affect women’s anxiety about their disease and how they manage their diagnosis.

Other researchers have proposed that we could draw on theories such as the Health Belief Model (Janz & Becker, 1984), minimization of the seriousness of risk, illness-specific beliefs, and optimistic biases (Weinstein, 1989) to help us to understand the many aspects of the experience of being diagnosed with osteoporosis (Reventlow et al., 2006; Rimes & Salkovskis, 2002; Rimes et al., 1999; Wallace et al., 2002). However, when the different aspects of our findings are integrated, they most closely relate to Leventhal’s Common Sense Model of Illness Representations, which states that beliefs are a major predictor of health behavior (Leventhal et al., 1997). This model has not been considered in relation to osteoporosis screening to date. Leventhal’s model proposes that cognitive representations can be grouped into five themes: identity, causes, curability/controllability, consequences (and the perceived seriousness of these), and timeline. People use two types of coping strategy to try to reestablish a state of equilibrium following their appraisal of the health threat; these are approach coping and avoidance coping (Leventhal et al.). We now discuss our findings using this model as a framework.

### Identity

Part of the cognitive process that occurs when someone tries to make sense of a diagnosis is the illness evaluation phase. This is when people try to understand and give meaning to their symptoms so they can subsequently build a mental model of their illness (Petrie & Weinman, 2006). The women in our study often described how they struggled to give an identity label to their disease. A significant barrier to being able to fully accept the diagnostic label was the invisibility and asymptomatic nature of the disease, as shown by the theme “lack of physical evidence creates doubt.” In the study by Reventlow et al. (2006), the women saw a visual image of their bone loss. They cognitively interpreted this to reconstruct their bodies as fragile and easily damaged. However, in our study the women received a verbal diagnosis from their GP. This seemed to make it difficult for them to give meaning to it, and some of the women expressed difficulty in accepting its accuracy because they could not see or feel any symptoms of illness.

As a population, the women held a cognitive representation of osteoporosis as being a painful disease, as shown in the subtheme “lack of pain means it isn’t serious.” They seemed to frequently draw on this belief to try to make sense of the accuracy and seriousness of the diagnosis in relation to their current bone health. They perceived pain as a symptom of advanced illness, and this did not seem to fit with the current asymptomatic experience of their bodies. The women often described how they felt healthy, and therefore believed that they could not have a serious illness. These reactions are consistent with views that when someone is diagnosed with an illness, they try to make sense of it by using their preexisting knowledge of that illness and their bodily experiences (Petrie & Weinman, 2006). The lack of visual evidence, as shown in the theme “osteoporosis is a routine medical condition,” meant that the women in our study had to draw on their beliefs about osteoporosis as a condition and try to make sense of it in relation to their current physical health. This was a struggle for them, especially given that many of them had only a basic understanding of osteoporosis. Being in a state of uncertainty caused by having an invisible and currently asymptomatic disease often led to them interpreting their diagnosis as only being a minor concern.

### Causes

The theme “osteoporosis as natural aging” suggests that women sought to make sense of their diagnosis by interpreting it in relation to their beliefs about the aging process. Many of the women expressed a fatalistic acceptance of the disease because they believed that their bone loss was inevitable as a result of the effects of aging on the body. This finding is consistent with those from other researchers who found that older people believed that the physical effects of aging were out of their control, and passively accepted them (Hurd-Clarke, Griffin, & The PACC Research Team, 2008).

### Curability/Controllability

The women in our study described feeling positive about the prognosis of their diagnosis because they seemed to view it as a treatable disease. A particularly distinctive theme was “the mediating role of medical care.” This suggested that any anxiety around the diagnosis was transient and largely ameliorated by medical intervention. The women were often highly complimentary about their GP and expressed great faith in their prescribing. Their belief in medication as the solution to their bone loss meant that they took their pills as instructed. This is positive, because it shows a good clinical outcome of screening in that treatment recommendations were being followed.

Even the few women who doubted the accuracy of their diagnosis or the benefits of taking medication still reported adhering to the medication routine. Although it might be argued that they could be misreporting their compliance, it is important to consider the generational experiences of this group in relation to how they perceive medicine as a profession. The women in this study were born between 1930 and 1941, growing up in a world where medicine became a miracle cure for illnesses that were previously terminal (Freidson, 2006). The dominant ideology of the time attributed power and prestige to doctors, and patients, by default, were submissive. As Freidson wrote, “This subordination is based on the assumption that a professional has such esoteric knowledge and humanitarian intent that he and he alone should be allowed to decide what is good for the layman” (p. xi).

The generational experiences of this group could help to put our findings into an appropriate context. The women’s views of the medical profession might explain why they all said they believed in the importance of medication and following their doctor’s instructions. None of the women had asked their GP about the purpose of their medication, and most of them demonstrated an unquestioning faith in its ability to stop their osteoporosis causing further deterioration. Although this compliance is clearly helpful in terms of future bone health, it can also mean that women have misconceptions about the role of their medication. For example, some women attributed incorrect meanings to the purpose of medication, such as seeing it as a cure for their osteoporosis rather than a way of slowing its progression. This could give them a false sense of security, because they might think that their bones are stronger than they are, and not take necessary precautions against falling.

In terms of controlling the disease through lifestyle changes, there was a widespread lack of knowledge about the benefits of weight-bearing exercise and diet on bone health. A few women described trying things to help themselves, but often mentioned interventions such as having a low-fat diet and swimming, neither of which are of optimal benefit to bone health (Wallace et al., 2002). Although some researchers have suggested that basic education about osteoporosis has a poor outcome on behavior change (Sedlak, Doheny, Estok, Zeller, & Winchell, 2007; Wallace et al.), other researchers have found a positive relationship between knowledge and bone-friendly behaviors (Satterfield, Johnson, Slovic, Neil, & Schein, 2000). The women in this study expressed motivation to help themselves (e.g., through staying active); therefore, it is possible that compliance with lifestyle changes could be high in this particular group of women if they had the appropriate education.

Ideas expressed in the “trust in the GP” subtheme gave a sense that the women’s cultural experience of the GP as an unquestionable authority meant they did not ask important questions about how their osteoporosis was being treated. The women who did not understand the meaning of their diagnosis, how it had been reached, or how accurate it was did not ask for more clarification. There was often an implicit assumption that the GP would tell them everything they needed to know, and that they did not need to seek additional information. Also, the women might not have known where to look for more help. This is a particularly important point given that many people now seek information about health issues from the Internet, but this generation might not be as computer literate (Morris, Goodman, & Brading, 2007). It is interesting to note that a number of the women asked the researcher questions about whether their understanding of osteoporosis was correct, and if there were other things that they could do to help themselves. It might be that the nature of the research evoked additional concern or curiosity in the women that they had not previously accessed. It might also be that they had not felt able or been given the time to ask such questions, given the frequently short nature of GP consultations in the United Kingdom.

### Consequences and Timeline

Although most of the women seemed to accept that osteoporosis was a disease which was irreversible, they did not particularly talk about it in terms of a timeline and the perceived consequences in the future. Their ideas about the effects of the disease seemed to be based on their present reality of living with the diagnosis. The women’s sense-making process in this area could be understood by the beliefs expressed in the curability/controllability section above, such as believing that osteoporosis is treatable. Their interpretations could also be understood by the ideas expressed in the themes “osteoporosis is a routine medical condition” and “lack of physical evidence creates doubt,” both of which express a sense that the asymptomatic nature of the current presentation of osteoporosis, coupled with the view that it is a common problem in older age, means that there is little to be concerned about. The effects of the disease on their lives at the time of the interviews were insignificant. Many women said that as long as they took care not to fall and break a bone then they would be okay.

One concern about the findings of our study was that the meaning women gave to their diagnosis seemed to be based on a lack of accurate knowledge about the disease and its potentially serious prognosis. Every woman was able to describe osteoporosis as increased bone fragility, but many also described misconceptions such as confusing it with osteoarthritis and viewing it as a minor health condition. This is consistent with research that has demonstrated that women who have no first-hand experience of osteoporosis generally have a poor understanding of it, and tend to underestimate its potential health impact (e.g., Backett-Milburn, Parry, & Mauthner, 2000; Richardson, Hassell, Hay, & Thomas, 2002).

### Coping Strategies

The women in this study generally presented as resilient and optimistic individuals. The theme “protecting the self from distress” demonstrated that they carried out a number of positive coping strategies to manage health-related anxiety that were consistent with a cognitive behavioral model (Rimes & Salkovskis, 2002). Avoidant coping strategies were also used. Future research might help us to understand how these work, and whether they are used interchangeably, because our findings only provide tentative ideas. There was a sense that women actively managed their diagnosis in a number of ways. The subtheme “the influence of the mind” suggested that women interpreted negative thoughts about illness as undesirable, because they believed that ruminating on their diagnosis would impact on both their physical and mental health. It seemed that they often told themselves to think positively about their health to take control of this feared negative outcome. Throughout the interviews, many of the women described other active coping styles toward their disease, such as taking their medication and taking precautions against falling.

The “keeping things in perspective” subtheme suggested that women also managed the emotional impact of their diagnosis by drawing on their ability to manage past adversity, and focusing on the moment rather than looking into the future. Wilkins (2001) proposed that women with osteoporosis who use such strategies are able to accept their diagnosis yet still maintain their self-concept of themselves as confident, strong, and capable of dealing with problems. Many of the women also described patterns consistent with an avoidant coping style, such as not allowing themselves to think about their diagnosis and expressing various degrees of disbelief in its accuracy. As has been argued, degrees of denial can operate as a useful defense mechanism which protects someone from emotional distress while facilitating them to carry out problem-focused coping (Evers-Kieboom, Welkenhuysen, Claes, Decruyenaere, & Denayer, 2000; Lazarus & Folkman, 1984). Another consideration, as Charmaz (1991) noted, is that denial might be from a lack of understanding about the disease rather than a refusal to accept the diagnosis.

Another factor that might have facilitated the women’s ability to cope with their diagnosis was the asymptomatic nature of their disease to date. Their ability and determination to continue with their daily lives and familiar routines might have given them a sense that their disease could be easily managed. It has been suggested that when people’s bodies are in a state of predictable health (e.g., low-level but manageable pain) and life stresses around them are minimized, they are able to focus on their daily lives and their health problems do not intrude on their enjoyment of their world. As a result, people feel able to continue with their usual routines and can minimize their illness (Olsson, Skär, & Söderberg, 2010). The women in our study often said that they found it easy to forget about their diagnosis unless something reminded them of it.

Given the lack of high levels of emotional distress among the women, it could be tentatively assumed that they had mostly attained a state of equilibrium according to Leventhal’s model (Leventhal et al., 1997). Whether this is a positive or negative adjustment needs to be assessed on an individual basis, but our findings suggest that most of the women had adapted well to their diagnosis, both emotionally and behaviorally. However, this might not necessarily be a positive finding, because research shows that patients often have limited medical knowledge, and the models they construct might be inaccurate (Martin, Rothrock, Leventhal, & Leventhal, 2003). It is important that patients have a correct understanding of the disease, especially because osteoporosis is often not symptomatic until it is in an advanced state, despite the fracture risk being present for many years beforehand (NICE, 2008). It is also important to consider that adjustment is an ongoing process. It has been suggested that emotional distress often does not arise until an illness becomes symptomatic (Gold, 1996). Therefore, Leventhal’s model could also be used to help us understand possible future reactions to the progression of the disease.

### Limitations

The data from this study are based on just 10 women from a single-center study in the United Kingdom, and IPA methodology does not aim to generalize to other populations. This would need to be investigated through further research. One particular problem was the lack of consistency in how GPs delivered the diagnosis, or how the women remembered this. Most of the women recalled being told that they had osteoporosis, “soft bones,” or “more fragile bones,” and then being given medication. It is unclear how much additional information they had, or the accuracy of their memory recall. Although this is a realistic clinical situation, it would have perhaps been more useful for the purpose of this research if every woman had been given the same information at the time of the diagnosis.

The role of the researcher (Joanne Weston) was important. Although the women knew that she was not medically trained, many approached the interview as if it was a medical consultation. It is particularly important given these findings to consider the possible effect of a power differential on the women’s responses, such as the high level of medication compliance reported. However, Weston did emphasize her lack of medical training, and maintained a stance of curiosity to encourage the women to talk freely about their feelings.

### Implications for Practice

A number of issues arose from our research which are particularly salient for clinical practice, especially in primary care settings. It might be beneficial for health care professionals involved in osteoporosis screening programs and GPs to consider the following:

- The theme “the mediating role of medical care” reveals the patient–doctor power dynamics that can be inherent in the medical system. Patients are often in a disempowered and vulnerable position (Fox et al., 2009); therefore, it might be particularly important for primary care professionals to check a patient’s understanding of his or her diagnosis before he or she leaves the consultation.
- The themes of “osteoporosis is a routine medical condition” and “lack of physical evidence creates doubt” suggest that the women struggled to understand the meaning and accuracy of the diagnosis and the implications for their health. Patients might benefit if primary care professionals give a diagnosis which includes sensitively explaining how a diagnosis has been made, and what the implications might be for their health.
- It could be helpful for patients to be told by their GP that it is common not to experience any symptoms or pain with osteoporosis unless it is at an advanced stage.
- Patients are likely to benefit from a follow-up appointment with their GP to receive education about diet and exercise, so they do not rely solely on medication to manage their osteoporosis.

## Conclusion

Osteoporosis is a health condition that is starting to attract more attention from health care providers (Medical Research Council, 2007). However, the results from this study show that it is imperative that we have a greater understanding of how patients might experience and adjust to receiving a positive result, so that we can promote optimal management of the disease. There is currently very little published research in this area, especially research focused on age-appropriate, population-based participants. The findings from our study suggest that screening did not cause significant emotional distress to the women; they were still living ordinary lives and their good relationship with medical care meant that they were adhering to medication recommendations. However, there are concerns about the management of the diagnosis, because the women often lacked understanding about osteoporosis as a disease. As a result, they were left with a lack of clarity about their condition regarding issues that they had not discussed with their GP. The findings also show that living with an invisible disease created some confusion in many participants, even though they had good psychological mechanisms to manage this, often using strategies consistent with a cognitive behavioral model.

Future research is needed to verify the generalizability of these results so as to add to our knowledge base. It would also be useful to develop the psychological understanding of these adjustment processes using appropriate theoretical models, such as Leventhal’s Common Sense Model of Illness Representations (Leventhal et al., 1997), as suggested by our study. Our findings provide tentative and useful insight into women’s experiences of receiving a positive result from an osteoporosis screening program.
